# Escape Rooms as a Learning Strategy for Special Education Master’s Degree Students

**DOI:** 10.3390/ijerph18147304

**Published:** 2021-07-08

**Authors:** Ana Manzano-León, José M. Rodríguez-Ferrer, José Manuel Aguilar-Parra, Ana María Martínez Martínez, Antonio Luque de la Rosa, Darío Salguero García, Juan Miguel Fernández Campoy

**Affiliations:** 1 Department of Psychology, University of Almería, 04120 Almería, Spain; aml570@ual.es (A.M.-L.); dariosalguero@ual.es (D.S.G.); 2 Department of Education, University of Almería, 04120 Almería, Spain; amm871@ual.es (A.M.M.M.); aluque@ual.es (A.L.d.l.R.); jfc105@ual.es (J.M.F.C.)

**Keywords:** special education, escape room, educative games, flow, motivation

## Abstract

Escape rooms and breakout are learning strategies that can facilitate motivation of learning through challenges. In these strategies, students must work as a team and use their reasoning, knowledge, and skills to solve puzzles and challenges related to the content of the curriculum, allowing them to solve the game in a limited time. The aim of this study was to determine the effects of the implementation of an escape room on classroom flow, academic performance, school motivation, and prosocial and antisocial behaviours with higher students in a Special Education master’s degree course. The quantitative results show a significant improvement in classroom flow, academic performance, and classroom climate, and a better score in prosocial and antisocial behaviours. The qualitative findings provide a better understanding of these results, and support the conclusion that the use of escape rooms is fun and motivating for students, and facilitates their learning achievement.

## 1. Introduction

One area of investigation in higher education is the use of active teaching strategies combined with ludic elements, such as gamification [1] and game-based learning [2]. The use of escape rooms is also beginning to be studied [3]. Escape rooms are immersive games played by small groups of three to eight people, in which participants are required to solve puzzles to escape from a room or, in the case of breakout, open a final chest.

The experience of the escape room has been a global phenomenon for almost a decade [4]. Escape rooms involve being “voluntarily” locked in a room, solving a series of riddles, unlocking locks, and finding hidden clues to escape. Using a variety of scenarios and challenges, the escape rooms create an experience that is simultaneously motivational and educational for the participants. To escape the room, players often open several four-digit coded locks. For this purpose, players must solve different puzzles to discover the code. They need to perform the task in a certain period, usually less than one hour [5]. Riddles are usually of the following forms: decrypt messages; find information in text; read text in a mirror; reveal invisible messages with UV light; search for items in strange places; combine parts; or activate a magnetic lock. The essential elements of an escape room are: Pattern: Escape rooms are built based on a pattern that can be linear (an orderly sequence of challenges), open (the final task is solved with the combination of the solutions of challenges of all teams), or multilinear (those in which you can simultaneously develop two or more lines of clues/puzzles throughout the game). Challenges, puzzles or tasks: These are the diverse elements whose resolution lead to the exit, or the discovery of a mystery, a cure, etc. Physical/online elements to solve tasks within an escape room: The most common are padlocks, puzzles, hidden codes, encrypted messages, riddles, and hidden objects. Clues: When the team of players fails to advance in the escape room, the Game Master (GM) can offer a clue to allow participants to continue moving forward, thus ensuring the group does not become frustrated. Depending on the type of escape room these clues must be obtained or requested directly from the master. The clues can be obtained via voice (calls, videos, walkie-talkies, audio signals, etc.), in person (either because the organizer is present or because they can respond through a device), or written (hidden, slipped under the door, projected, etc.). Narrative: an escape room is more than a sequence of puzzles to open boxes; the narrative is a thread by which all of the challenges are related. Educational escape rooms are not always designed with a narrative. The design is simplified if the narrative does not act as a guiding thread that relates all of the challenges; however, this is less immersive because the narrative itself is part of that initial impact that motivates the player to undergo the experience.

Real-life escape rooms are a new game genre based on live action role-playing games, treasure hunts, and online escape rooms [6]. Most escape rooms are purely recreational; however, educational escape rooms are becoming more popular with professional programs to involve students in their learning environment, and encourage collaboration and the development of social skills [7]. The results of recent studies show that games and the use of escape rooms have been effective in involving students in the learning process and helping them retain information [8]. Dietrich [9] reinforces this idea because his study shows that an escape game in the classroom encourages students to discover scientific concepts in a team environment and playful manner, and provides opportunities to develop adaptive and receptive skills, compete with and against their classmates, show their individual skills, interact with each other, and experience moments of discovery and victory. Problem solving and critical thinking can be highlighted among the skills that can be developed with the use of escape rooms in the classroom. Critical thinking consists of being able to understand thoughts, make sense of ideas, and make logical decisions [10]. The escape rooms pose different challenges and tasks that make students question and evaluate their ideas, and solve problems.

Escape rooms can generate intrinsic motivation in the players. Other advantages include favoring learning, improving attitudes and social skills, and involving students with the subject and teamwork [11,12]. Ultimately, escape rooms could provide an exciting engagement for higher education programs.

Studies have shown that playful learning experiences generate motivation and commitment among most participants [13]. Game-based learning can involve students in a learning activity, thus achieving high levels of commitment (concentration, interest, and enjoyment). This can be accomplished by increasing the levels of challenges and skills during the game [14]. Ensuring the game is a challenge, therefore, is an especially strong predictor of learning outcomes. The motivation to undertake challenging tasks is related to classroom flow. Flow is defined as a state of total immersion and fusion of action and consciousness [15], and is associated with positive emotional, motivational, and cognitive experiences [16]. The degree to which students enjoy a subject and their degree of motivation can be predictors of their results [17].

The use of gamification and playful strategies improves motivational learning because it allows students to experience and discover, while practicing skills and learning in a playful manner [18]. Other benefits of using escape rooms may be as a potential avenue for co-workers, classmates, or friends to explore and improve their collaborative skills, socialize with others, and develop team morale [19]. Collaborative learning is an effective approach to improve student outcomes. These tasks allow small groups of students to collaborate and share perspectives, discuss points of disagreement, question and understand the points of view of others, solve complex problems, and reach agreements [20]. In addition, the development of collaborative skills can be positively related to prosocial behaviours [21], defined as a set of behaviours whose objective is to establish socially empathic and cooperative relationships. In contrast, antisocial behaviours are behaviours intended to harm and distress other people [22]. Other playful strategies such as gamification facilitate the creation of a relaxed climate, reducing disruptive behaviours and generating a sense of control and responsibility towards learning [23].

There is emerging evidence that escape rooms and breakout are being used in university education, and that this can have a positive impact on motivation and the relationships between students [3,8,24]. However, this approach is not yet a consolidated learning strategy for master’s students. Our study aimed to fill this knowledge gap and evaluate the impact of escape rooms on learning, motivation, classroom climate, and overall satisfaction of the experience of Special Education master students. Specifically, the objectives of this study were: (1) To determine the effectiveness of an intervention through an escape room in relation to classroom flow, academic performance, classroom climate, and the prosocial and antisocial behaviours of the students; (2) to explore the relationship between the use of the escape room and the learning and motivation of the students.

## 2. Materials and Methods

### 2.1. Participants

The context of this study was the implementation of an educational room escape in the subject “Psychic Disability” of the Master of Special Education of the University of Almería during the academic year 2018/2019. A total of 26 female and 4 male students, with ages between 21 and 44 years (*M* = 26.06; *SD* = 7.12), participated in the experience and voluntarily completed the questionnaires and interview.

To select the participants, a convenience sample was chosen. The inclusion criterion for the experimental group was a willingness to participate in the escape room organized by the teaching staff and researchers. Participants received information about the project and gave written informed consent. Prior to data collection, the students were informed about the nature of the study and were assured of their anonymity. The final evaluation was carried out by the class teachers according to written instructions. Students completed the questionnaire during a regular class.

This study complied with the recommendations of the American Psychology Association. The entire process was conducted in accordance with the Helsinki Declaration [25]. All the participants also gave their oral and written informed consent. Ethics approval was obtained from the Research Ethics Committee of the University of Almería (Ref. UALBIO 2021/01).

### 2.2. Design of Escape Room

**Figure 1:**
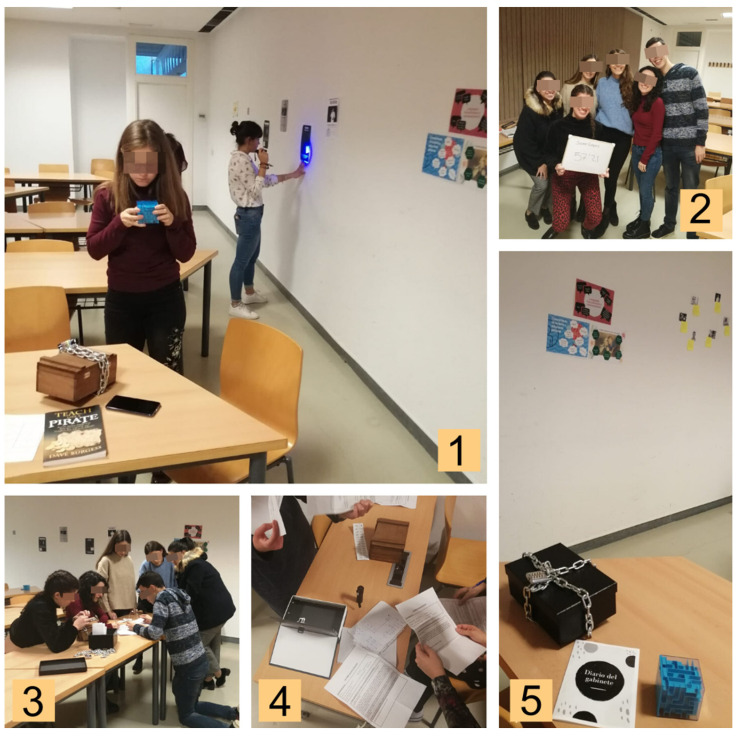
The escape room design and implementation. (**1**) Students distributed around the room looking for clues to exit; (**2**) final photo after the escape room; (**3**,**4**) students working cooperatively to solve challenges of the escape room; (**5**) some of the elements of the escape room.

In the subject “Psychic Disability” of the Master of Special Education at the University of Almería, it was proposed to experience an escape room in the penultimate session of practical classes. The entire group was separated into 6 groups of 5 students. In the days prior to its completion, students were urged to review the documentation and content of the subject for “a complementary activity”. Until that day, the students were not informed that they would experience the escape room, and the escape room was a large surprise. Two external professionals were masters of each other’s game. Both professionals had more than 5 years of experience in the organization and development of playful experiences such as escape rooms, and coordinated with the teacher of the subject so that the quests had a didactic purpose related to the subject. These rooms were thematically decorated with books, posters, photographs, and materials related to the challenges of the escape room. In each of the classrooms, the students of each team were “locked up” with all of the material necessary to carry out the activity, and were prevented from contacting the other team, which played simultaneously (Figure 1).

Before starting the activity, the game masters explained the rules necessary to perform the escape room. The rules were simple: all of the clues are at players’ fingertips, it is not necessary to use force or break anything, and the clues and tasks only have one use; for example, a code will only open a lock, it will not serve to open others. In addition, the game masters explained the narrative situation through which they entered the escape room, to contextualize the situation and create a suitable environment. The escape room narrative consisted of a good practice pedagogical cabinet in which a group of educators had been kidnapped by a group of “bad pedagogical practices” professionals. Once the students entered the room, they listened to an audio where that bad group had also locked them up so they wouldn’t tell their secrets. Subsequently, the countdown appeared and each team, cooperatively, had to solve the five challenges of the escape room. One of these was purely playful and involved removing a ball from a box. However, the other four challenges (two boxes with padlocks and two QR code locks) were related to the contents of the subject, and participants were required to look for clues in the room about how to diagnose or intervene with students with psychic disabilities.

The objective of this educational escape room was to review the content of the Psychic Disability course and for the students to learn to work cooperatively. The proposed educational challenges were:

(a) Solve a quiz to defuse a bomb, relating the names and definitions of Down syndrome, autism spectrum disorder, intellectual disability, and mental disorder.
(b) Correctly answer 4 single-solution questions regarding the curriculum content, with 5 alternative answers, to open a physical lock.
(c) Correct 10 educational reports for students with mental disabilities, identify those that are incorrect to be able to discard them, and identify the appropriate schooling modality of the 4 correct reports to indicate the final answer of the door code.

The Game Masters encouraged participants to work as a team, find clues around the class, and make the most of the time among all of the members. If they observed that the students were frustrated or asked for a clue, they offered assistance so that the game had the appropriate pace of challenge and difficulty. It is important to note that most of the students had no previous experience of a conventional or educational escape room. Once the group escaped, a photo was taken in the classroom with their escape time and their team’s name.

The total escape room time for each group of students was between 40 and 100 min, depending on how long it took them to solve the case. It took 20 min to reorder the clues on the stage between the different groups. The use of two professionals allowed two identical escape rooms to be replicated in two university classrooms.

### 2.3. Instruments

A unique case study was conducted with a mixed methods approach in a parallel convergent design [26] to examine the relationship between escape room design and student development. This design implies that the researcher uses quantitative and qualitative techniques at the same time during the same phase of the research process. The priority of the methods is the same in both approaches, which are separated and independent during the analysis. The results are finally combined during a joint interpretation.

Quantitative data were collected in a pretest-posttest group design with the following questionnaires:

- Brief Inventory of Optimal Experiences [27] was applied. The inventory comprises 9 items, from which a total score of optimal experience in the evaluated activity is obtained. The 9 items have 5 response options from totally agree to totally disagree. Cronbach’s Alpha values were 0.864.
- The Short Class Climate Scale [28] which has 11 items, divided into 2 dimensions (Group cohesion and Group leadership) and 5 subdimensions (Satisfaction and involvement, Peer cohesion, Teacher–student relationship, Order and organization, and Orientation to the task) ranked on a 4 point Likert scale, which presents 4 possibilities of response: never, sometimes, frequently, and always. A Cronbach’s alpha coefficient of 0.83 was obtained for the scale.
- The scale of prosocial antisocial behaviours [29]. This instrument has 20 items, divided into 2 factors (Prosocial Behaviors and Antisocial Behaviors) and 4 subfactors (Prosocial Teammate, Prosocial Opponent, Antisocial Teammate, and Antisocial Opponent) ranked on a 5-point Likert scale, from strongly disagree to strongly agree. Cronbach alpha values of the factors and subfactors were greater than 0.70 and gender-invariant. For this study, the Prosocial Teammate and Antisocial Teammate subscales were used.
- Five ad-hoc questions of the subject with four answer options, one correct and three incorrect. An example of the question was: “How do teachers evaluate participation, interaction and socials roles in students with severe intellectual disability?”. Students were asked to complete the questionnaire before beginning (t1) and at the end (t2) of the escape room.

### 2.4. Data Analysis

To examine the results of the study, a mixed methodology was performed. First, a *t*-test was performed for related samples because the students were measured before and after developing the escape room. To complete this analysis and quantify the effect size, Cohen’s d was used. The SPSS 25.0 statistical package (IBM, United States) was used for quantitative data analysis. Qualitative data were collected in semi-structured interviews [30] of students who participated anonymously and freely expressed their opinions on the escape room project. All information was organized for analysis and treatment through the Atlas.li software (version 8.4.2, ATLAS.ti Scientific Software Development GmbH, Berlin, Germany). The analysis process, based on the grounded theory [31], was carried out through three processes: open coding, axial coding, and selective coding [32].

After collecting the transcript, open encoding was performed first. This consists of a careful examination of the data to identify the meanings of the students’ responses. To ensure the validity and thoroughness of the analysis, two researchers, separately, recorded the first open codes that expose the thoughts and meanings of the surveys. Then, in axial coding, these two researchers combined their codes and unified or divided one of them if necessary. In the case of doubts about the interpretation, the help of a third investigator was requested. In this phase, the categories and subcategories of the stories were related. Once the categories and subcategories were collected in the axial coding, selective coding was carried out. In this phase, the categories found in the stories were organized into relationship networks or concept maps. Structural networks or flow diagrams graphically represent possible structures or systems of relationships between categories or codes. In this network, the interpretations are made explicit, and it allows reviewing of all the elements that can support one or another argument or conclusion [33]. This phase was also carried out based on the discussion of two researchers and, in the case of disagreement, a third researcher was contacted.

## 3. Results

Table 1 shows the results related to the study variables before and after the escape room. As can be seen, there were statistically significant improvements in the variables flow, academic performance, and classroom climate, with the exception of the task orientation subfactor. There were also improvements, although not statistically significant, in prosocial and antisocial behaviours; there was an increase in the means in prosocial behaviours and a decrease in antisocial behaviours between pre- and post-test. The size of the effect, calculated using Cohen’s *d*, showed a very strong effect on flow, academic performance, peer cohesion, and teacher–student relationship. There is also a strong effect in order and organization, a moderate effect in satisfaction and involvement, and a low effect in the remaining variables.

**Figure 2:**
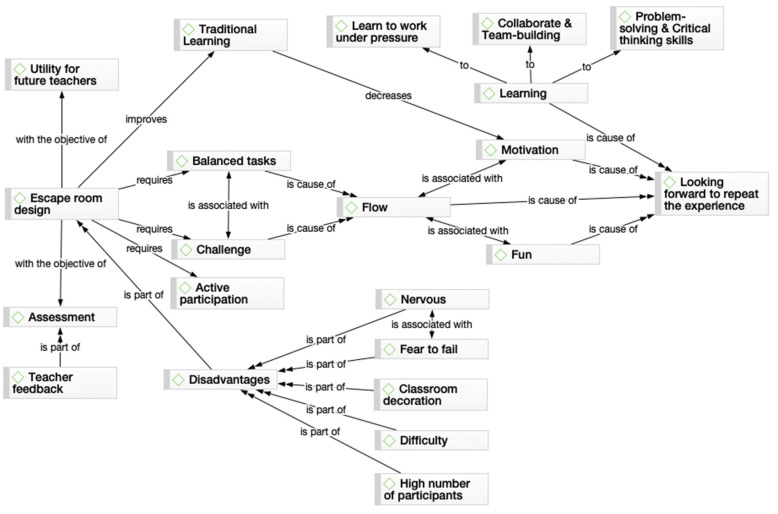
Network diagram of student’s experiences about the educational escape room.

After the analysis of the interviews conducted with the students, the following categories were identified (Table 2, Figure 2). The first number in the following tables represents the name of the student, anonymized as S (which stands for student) and a number (which was chosen for each student in a linear order).

### 3.1. Escape Room Design

The escape room design includes all of the playful and educational elements that were taken into account when preparing the activity. When designing escape rooms, special emphasis is placed on the link between puzzles, challenges or puzzles to solve, and narrative. In education, the challenges introduced should not only be fun and related to the narrative, but also have a didactic purpose that has an impact on students and helps them learn new skills or reinforce acquired content. The results of the interview analysis show that the students found that the design of the escape room had balanced tasks (20% of the student statements), and the challenge (43.4% of the student statements) was satisfactory and exciting. Another interesting aspect of the escape room was the possibility of evaluating curricular contents from the tests carried out in a playful manner with the objective of offering feedback on their abilities. A share of 30% of the students mentioned in their interviews that the evaluation of their abilities was very positive because they did not feel pressured and were having fun, and 6.7% mentioned that the presence of the teacher in the classroom encouraged them to solve the tests. Students realized the relevance of the use of escape rooms in education because 20% of them talked about its usefulness in their future elementary classes.

### 3.2. Experience

In educative escape rooms, the experiential aspect is vital for motivating students because it aims to provide a memorable learning experience. To be a credible experience, the narrative and the tasks must be connected to the narrative and the message it is trying to convey. In addition, these tasks must meet flow conditions such as clear objectives, feedback, and linkage to the game. Within the category of experience, the effects mentioned by the students focused on positive feelings, emotions, and results, which were divided into the subcategories of flow, motivation, fun, and looking forward to repeating the experience. For most students (90%), the escape room was fun. They mentioned that it was different and more fun than traditional classes, and consequently more motivating (83.4% of the students’ statements). The design of the challenge and the need to collaborate was a very positive and motivating factor for the students. In addition, the level of challenge (neither too easy nor too difficult) was adequate, and the goals were clear for the students. Thus, the experience was enriching with a high flow (83.4% of the students’ statements).

### 3.3. Learning

The main objective of the educational escape rooms is to create a creative learning environment, which can be designed for any educational level, and uses the characteristics of the escape room’s design to incorporate specific educational elements and purposes. As a result of the interview analysis, the category of learning includes the acquisition and improvement of educational and interpersonal skills. Students indicated that their own capacities, such as problem-solving and critical thinking (16.7%), collaborating and team building (50%), and learning to work under pressure (13.4%), were put into practice during the escape room. Furthermore, it was mentioned again in this category that the learning acquired in the escape room seemed more significant than that in traditional education.

### 3.4. Disadvantages of Escape Room

During the interview students were asked to mention the escape room factors they did not like or could be improved. It should be noted that 60% of the students considered that there was nothing to improve. However, any educational activity has a range of improvement. Within this category, the results found from a small portion of the students related to nervousness and fear of failure during the test (3.4%). These sensations were produced due to the limited time of the escape room and the feeling of being watched by peers during the experience. Another participant mentioned that the number of students (7/8 per group) was very large for the class size. Finally, the difficulty was mentioned (13.4% of the students’ statements). The moment of feedback inside the escape room was key to obtaining the flow environment because if the clues arrived too early, a balanced challenge would not be achieved, whereas if the clues arrived too late, the participants would feel frustrated because it will seem too difficult.

## 4. Discussion

Educational escape rooms are gradually being established in formal education settings, where it has been observed that the experiences enhance the development of skills and competences for students, in addition to being a motivating element in their education. To the best of our knowledge, this type of game has not been previously used in education master studies, although it has been used in other university degrees [34,35]. According to the students consulted, the escape room was fun, it helped them to review the contents of the subject, and they were able to enjoy working in teams and active learning. Students also mentioned that more learning strategies of this type should be used during the master’s degree. The impact of gamified activities and escape rooms in university studies, and the desire of students to apply more games of this type in other educational subjects, demonstrate that these teaching strategies are suitable for motivating students. This finding is supported by the findings of previous studies, such as that of Nicholson [36].

Our study showed the effectiveness of escape rooms in relation to classroom flow, and obtained statistically significant improvements. These results coincide with other studies such as that of Shernoff [37], who states that for students to reach a state of flow it is necessary to generate experiences that simultaneously provoke concentration, interest, and enjoyment. This is based on an optimal classroom flow in which the aspects of challenge (concentration) and play (enjoyment) are favored. This allows the experience to be intrinsically significant and fulfils a preventive function regarding the negative consequences for learning [38]. This is reinforced by the qualitative data, which indicated that the students perceived the escape room as a challenging cognitive task that they could enjoy as a team and, consequently, this allowed a high level of classroom flow. There were also significant improvements in the ad hoc academic performance test. This indicates that the use of escape rooms can improve the acquisition of curriculum content. Our results reinforce previous research [11,39,40,41], which found that escape rooms can be a fun and motivating teaching–learning strategy to reinforce and evaluate the curricular contents of university teaching.

The low cost of escape rooms is another positive aspect compared to the implementation of learning projects with video games, virtual reality, or serious games in which specific software and hardware must be developed and maintained. The design of this escape room only required the purchase of four boxes, chains, and four padlocks with combinations, in addition to making two digital locks with QR codes. However, to ensure the room had greater ambience and looked more like a conventional escape room, a larger budget may be required.

Regarding prosocial and antisocial behaviours and the classroom climate, no significant differences were found. However, improvements were found after the escape room. This result can be explained because the activity was only undertaken in one session and these variables need more stimulation time for these changes to occur. Previous research, such as that of Tobón [42], supports the idea of a profile of social responsibility in students, within which prosocial and antisocial behaviours exist, based on values and moral development. This profile would be based on university education and articulated with psychosocial factors, such as age, family structure, and economic and political factors that are transversal in the development of prosocial, collectivist, and moral behaviours. Considering these data, we conclude that a specific activity of one or several days is not enough to be able to achieve significant changes in prosocial and antisocial behaviours. The climate in the classroom is a multifaceted concept. Alonso-Tapia, et al. [43] studied how teacher action patterns can have an effect on the overall classroom climate, and how the set of interactions between students also exerts an important influence on positive interactions. Students and peers provide personal validation and emotional support, help solve problems, and help to develop a perspective and empathy. These characteristics serve as the basis for cooperative, prosocial, and non-aggressive behaviours. There is a lack of empirical research on the impact of escape rooms and other recreational strategies on the relationship between teachers and students, and that of students among themselves. However, we consider it remarkable that better scores were obtained for the classroom climate and prosocial and antisocial behaviours after the intervention, and that it is necessary to study its use with a greater number of implementation sessions.

From the completion of this study, it is considered that educational gamification can achieve multiple benefits: it is an innovative learning strategy that encourages active student learning and pursuit of engagement; it also stimulates the curiosity and participation of students, provides opportunities to achieve a well-designed classroom flow, and creates a positive classroom climate and allows meaningful learning. From the evaluation, we can conclude that that educational escape rooms have a complementary function to traditional didactic teaching as an innovative and motivating learning strategy. Most of the interviewed participants claimed that the escape room was very motivating and fun, and served to consolidate their knowledge about the subject. The students also noted the potential use of the escape rooms to evaluate their knowledge in a playful manner. However, it should be noted that a small portion of the participants felt stressed or were afraid to fail during the activity.

Finally, the limitations of this study are mentioned. First, there was a lack of a control group with which to compare the variables. Although there are positive changes within the same group, it would be interesting for future research to evaluate the effectiveness of the escape room with respect to a control group that carried out traditional classes in the variables studied with validated instruments, and the performance of the complete subject through a school performance test. Second, it is also recommended to increase the study sample to generalize the results.

Another limitation is the lack of control for possible confounding variables (individual dispositions or interpersonal skills of the people involved in the study) that can affect the manner in which they participate in an escape room. In future research, these variables could be studied descriptively to determine if they act as a predictor of enjoyment or rejection of these playful experiences.

Regarding the design of the escape room, to make it more functional and have greater possibilities of replication in other university groups, it would be possible to create an open room escape system combined with online tools. One of the limiting factors of the escape rooms is that the size of the team must remain relatively small to optimize collaboration and commitment [44]; however, ordinary classrooms of universities have large enrolments of more than 100 students. The author made an escape room with that number of students, creating a blended environment. As a future line of research, it is proposed to carry out a longitudinal study of repeated measures with a control group and an experimental group to explore the long-term effects of the use of playful strategies on academic performance, educational flow, school motivation, classroom climate, and prosocial behaviors. Another future line of research could be to study whether there are differences in these variables, using other playful strategies such as gamification or game-based learning, in addition to whether there are statistically significant changes depending on the time of application of the programs.

## 5. Conclusions

In conclusion, the use of escape rooms in university education can have benefits in the motivation, flow, and acquisition of student knowledge, and can be combined with other strategies of active learning and traditional learning. Limited research has been undertaken on the use of recreational strategies in education students. Thus, significant information could be obtained about the development of key teaching skills, such as educational innovation, teamwork, and collaboration, with this type of teaching strategy.

## Figures and Tables

**Table 1:** Pre-test and post-test differences between the experimental group.

	M	SD	*t*	*df*	*p*	*d*
Pre-Flow	30.89	3.76	−6122	28	0.000	1.53
Post-Flow	36.89	4.08				
Pre-Academic performance	4.13	0.78	−5537	28	0.000	1.46
Post-Academic performance	4.96	0.18				
Pre-Satisfaction and involvement	6.65	1.36	−2862	28	0.008	0.57
Post-Satisfaction and involvement	7.31	0.89				
Pre-Group cohesion	6.10	1.44	−6425	28	0.000	1.31
Post-Group cohesion	7.58	0.68				
Pre-Teacher-student relationship	6.89	1.26	−3932	28	0.001	1.02
Post-Teacher-student relationship	7.86	0.44				
Pre-Order and organization	6.13	1.09	−3271	28	0.003	0.72
Post-Order and organization	6.86	0.91				
Pre-Task Orientation	8.51	1.45	−1870	28	0.072	0.40
Post-Task Orientation	9.24	2.11				
Pre-Prosocial behaviours	17.89	3.25	−711	28	0.483	0.17
Post-Prosocial behaviours	18.44	3.18				
Pre-Antisocial behaviours	7.24	3.93	0.725	28	0.474	−0.18
Post-Antisocial behaviours	6.55	3.83				

**Table 2:** Qualitative results after implementing the escape room.

Main Category	Sub-Category	Number of Statements	Examples of Statements
Escape room design	Balanced tasks	6 from 30, 20%	I liked having to use logic to get through the tests (S4)
	Challenge	13 from 30, 43.4%	The satisfaction and excitement produced by passing tests and finding new challenges (S9)
	Active participation	2 from 30, 6.7%	I found it a very interesting activity because I had not done it before. I have been able to put into practice what I learned in class and participate and interact with each of my teammates (S20)
	Assessment	9 from 30, 30%	A very good option to evaluate what we really know about the subject, because we didn’t feel evaluated and we had fun (S29)
	Teacher feedback	2 from 30, 6.7%	I would give it a 10 out of 10 and the teacher’s presence was very good, and he inspired confidence, as he continually encouraged us (S18)
	Utility for future teachers	6 from 30, 20%	I think it is a didactic experience that can work very well in the classroom, I would like to learn more about it to prepare classes for elementary students (S30)
Experience	Flow	25 from 30, 83.4%	I thought it was an enriching experience and constructive in strengthening my learning and researching other knowledge that was not so clear through overcoming challenges (S8)
	Motivation	25 from 30, 83.4%	Very interesting and motivating to apply in education (S11)
	Fun	27 from 30, 90%	A different way of transmitting knowledge and at the same time fun. A change from the normal class routine (S10)
	Looking forwards to repeat it	11 from 30, 36.7%	Well, I think that this type of activity should be used to learn concepts of various activities, as we know, what we experience, we learn better. I consider it one of the best activities I have done in all my university studies. CHAPEAU! (S27)
Learning	Problem-solving and Critical thinking skills	5 from 30, 16.7%	I liked the way in which content is working through puzzles (S15)
	Collaborate and Team building	15 from 30, 50%	It was an incredible experience, what I liked most was the fellowship, as we helped each other, the interest we put in solving the riddles, the use of all our knowledge... In short, the good atmosphere that has been created (S1)
	Learn to work under pressure	4 from 30, 13.4%	Very interesting, something new for me, although I felt a little pressure at the beginning, we were able to escape from the room (S3)
	Better than traditional learning	4 from 30, 13.4%	I would like it to be used more as a methodology than traditional education (S5)
Disadvantages of escape room	Nervous	1 from 30, 3.4%	It made me nervous that they could watch me while I was doing the tasks (S4)
	Fear to fail	1 from 30, 3.4%	I am not in favour of the escape room, due to the limited time and fear of failure, they are stressful for me (S6)
	Classroom decoration	5 from 30, 16.7%	What I liked least is the decoration of the classroom, the only thing that could be improved (S12)
	Difficulty	4 from 30, 13.4%	Some tasks were difficult (S2)
	High number of participants	1 from 30, 3.4%	It would improve the size of the classroom and the number of participants (S14)

Note: Chapeau is a generic expression that is used as appreciation or respect in France and other countries of Europe.
